# Several clinical interests regarding lung volume reduction surgery for severe emphysema: meta-analysis and systematic review of randomized controlled trials

**DOI:** 10.1186/1749-8090-6-148

**Published:** 2011-11-10

**Authors:** Wei Huang, Wen R Wang, Bo Deng, You Q Tan, Guang Y Jiang, Hai Jing Zhou, Yong He

**Affiliations:** 1 Thoracic Surgery Department, Institute of Surgery Research, Daping Hospital, Third Military Medical University, Chongqing, P.R. China

**Keywords:** LVRS, emphysema, meta-analysis, systematic review

## Abstract

**Objectives:**

We aim to address several clinical interests regarding lung volume reduction surgery (LVRS) for severe emphysema using meta-analysis and systematic review of randomized controlled trials (RCTs).

**Methods:**

Eight RCTs published from 1999 to 2010 were identified and synthesized to compare the efficacy and safety of LVRS vs conservative medical therapy. One RCT was obtained regarding comparison of median sternotomy (MS) and video-assisted thoracoscopic surgery (VATS). And three RCTs were available evaluating clinical efficacy of using bovine pericardium for buttressing, autologous fibrin sealant and BioGlue, respectively.

**Results:**

Odds ratio (95%CI), expressed as the mortality of group A (the group underwent LVRS) versus group B (conservative medical therapies), was 5.16(2.84, 9.35) in 3 months, 3(0.94, 9.57) in 6 months, 1.05(0.82, 1.33) in 12 months, respectively. On the 3^rd^, 6^th ^and 12^th ^month, all lung function indices of group A were improved more significantly as compared with group B. PaO2 and PaCO2 on the 6^th ^and 12^th ^month showed the same trend. 6MWD of group A on the 6^th ^month and 12^th ^month were improved significantly than of group B, despite no difference on the 3^rd ^month. Quality of life (QOL) of group A was better than of group B in 6 and 12 months. VATS is preferred to MS, due to the earlier recovery and lower cost. And autologous fibrin sealant and BioGlue seems to be the efficacious methods to reduce air leak following LVRS.

**Conclusions:**

LVRS offers the more benefits regarding survival, lung function, gas exchange, exercise capacity and QOL, despite the higher mortality in initial three postoperative months. LVRS, with the optimization of surgical approach and material for reinforcement of the staple lines, should be recommended to patients suffering from severe heterogeneous emphysema.

## Introduction

Emphysema is a chronic and progressive disease, characterized by permanent impairment of pulmonary terminal airway, hyperinflation of parenchyma and loss of elastic retraction. The shortness of breath, poor exercise tolerance and impaired health status will occur on the final stage of emphysema [1-3]. Thus far, the conservative medical therapies (antibiotics, bronchodilators, systemic corticosteroids, home oxygen therapy, pulmonary rehabilitation) still remain to be symptomatic treatment rather than always due to failure to improve elastic recoil of lung issue [2-5]. Lung volume reduction surgery(LVRS), which was initially introduced in 1957 by Brantigan [6] and developed by Cooper in 1993 [7], resects diseased and non-function pulmonary issue in order to ameliorate lung function, exercise capacity and health status, by(1) increasing pulmonary elastic recoil, therefore increasing expiratory airflow rates, (2) reducing the degree of hyperinflation, therefore improving mechanics of diaphragm and chest wall, (3) reducing heterogeneity, (4)increasing work of breathing, and improving of alveolar gas exchange [8].

Although numerous studies have addressed the patients with severe emphysema can receive benefits from LVRS, some physicians remain routinely reluctant to recommend LVRS to the suitable patients due to the insufficient published Randomized Clinical Trials (RCTs) evaluating surgical risks and long term sequels [9-12]. Besides, there are controversial points regarding the efficacy and safety of two approaches for LVRS [median sternotomy(MS) vs video-assisted thoracoscopy surgery (VATS)] [13]. In addition, various materials have been utilized to prevent air leak which is one of the most crucial risk factors for LVRS [14,15], but the efficacy should be assessed immediately. Herein, we performed a meta-analysis of RCTs published in the past 11 years for the sake of evaluating safety, short-term efficacy and long-term sequel of LVRS. And we conduct the systematic review of two approaches (MS vs VATS) and the materials (bovine pericardium for buttressing, autologous fibrin sealant and BioGlue) for LVRS.

## Materials and methods

We used systematic methods to identify relevant studies, assess study eligibility, evaluate methodological quality, and summarize findings regarding postoperative clinical outcomes.

### Data sources and searches

Medline and manual searches were performed by two investigators independently and in duplicate to identify all published RCTs during from 1999 year to 2010 year that addressed the issue of LVRS for emphysema.

The Medline search was done on Pubmed (http://www.ncbi.nlm.nih.gov), one set was created using the medical subject headings (MeSH) term 'pneumonectomy' (18249 citations, March 31^st^,2011) and another was created using the MeSH term 'pulmonary emphysema' (12953 citations, March 31^st^, 2011). Combining the two sets with the Boolean 'and' function yielded 1006 citations, This set was limited by the publication type 'randomized controlled trial' to give 36 citations in English. Manual searches were then done by reviewing articles cited in the reference lists of identified RCTs, and also by reviewing first author's article.

Eight published RCTs [2,12,16-21] were identified regarding LVRS vs conservative medical therapies (table 1). Among the eight RCTs, Pompeo's article [17] and Mineo's article [20] were from the same trial. Pompeo's study [17] presented mortality, but "mean ± SD" of lung function was missing, wheraes it was presented in Mineo's study. Therefore, we included both of the aforesaid articles. We did not include unpublished data because of the limited number of RCTs, trials were not excluded because of trial quality (design) or insufficient number of patients. A trial quality score was assigned (scale of 1-5) according to the method of Jadad et al [22]. One investigator screened the articles and identified article abstracts for full review.

**Table 1:** Summary of RCTs on conservative medical therapy and LVRS

Author	Year published	Cases (N)	Imaging diagnosis	Cases underwent conservative medical therapy (n)	Cases underwent LVRS(n)
Criner, G.	1999	37	CT scan	18	19
J[16] Geddes, D[12]	2000	48	CT scan	24	24
Pompeo, E. [17]	2000	60	CT scan	30	30
Fishman, A [18]	2003	1218	CT scan	608	610
Goldstein, R. S [19]	2003	55	CT and V/Q scan*	28	27
Mineo[20]	2004	60	CT scan	30	30
Hillerdal, G. [2]	2005	106	CT and V/Q Scan*	53	53
Miller, J. D[21]	2005	93	CT and V/Q Scan*	58	35

*: V/Q scan, also known as lung scan, which evaluates both the Ventilation and Perfusion of the lungs using scintigraphy and medical isotopes. Q is the symbol for perfusion which represents the movement of blood through the arteries that supply the lung.

One RCT [13] regarding comparison of two approaches for LVRS (MS vs VATS) was obtained. And three RCTs [14,15,23] were available evaluating clinical efficacy of using bovine pericardium for buttressing, autologous fibrin sealant and BioGlue, respectively.

### Data abstraction

Two investigators abstracted the following information from the eligible articles without blinding: author, location of study site, journal, year of publication, study design, number of patients, demographic characteristics, clinical outcomes, and follow-up period. In all of the included articles, patients underwent LVRS. Major clinical outcomes for quantitative data synthesis included postoperative mortality, lung function, gas exchange (PaO2 and PaCO2), DLCO, 6MWD. Disagreements were resolved by consensus review with a third investigator.

### Statistical analysis

#### Test- and study-specific estimates

Major postoperative outcomes are defined in the index tests as follows:(1) Postoperative mortality in the 3, 6 and 12 months.(2) Postoperative Lung function on the 3^rd^,6^th ^and 12^th ^month including FEV1, FEV1%, RV% and TLC%.(3) gas exchange and DLCO% on the 6^th ^and 12^th ^month.(4) Postoperative 6MWD on the 3^rd^, 6^th ^and 12^th ^month.(5) QOL: We performed systematic review, instead of meta-analysis of QOL in the RCTs due to the different evaluating criterion including the Sickness Impact Profile (SIP) scoring system [16], the 36-item short-Form Health-Related Questionnaire(SF-36) [2,12,20,21], the Nottingham Health Profile (NHP) [20], the St George's Respiratory Questionnaire (SGRQ) [2,20], Quality of Well-being scale [18] and the Chronic Respiratory Questionnaire (CRQ) [19,21].

### Meta-analysis model

A fixed-effect model was applied when the P values of test for heterogeneity is more than 0.5. A random-effects model was used as it provided conservative confidence intervals for postoperative outcomes between study variability (*P *< 0.05). Odds ratio or weighted mean difference was the principal measure of effect. They were presented as a point estimate with 95% confidence intervals and *P *values in parentheses. Review Manager 4.2.2 (The Cochrane Collaboration. Wintertree Software inc, Canada) statistical software was used. Publication bias could not be properly assessed because there were insufficient RCTs to construct a funnel plot.

## Results

**Figure 1:**
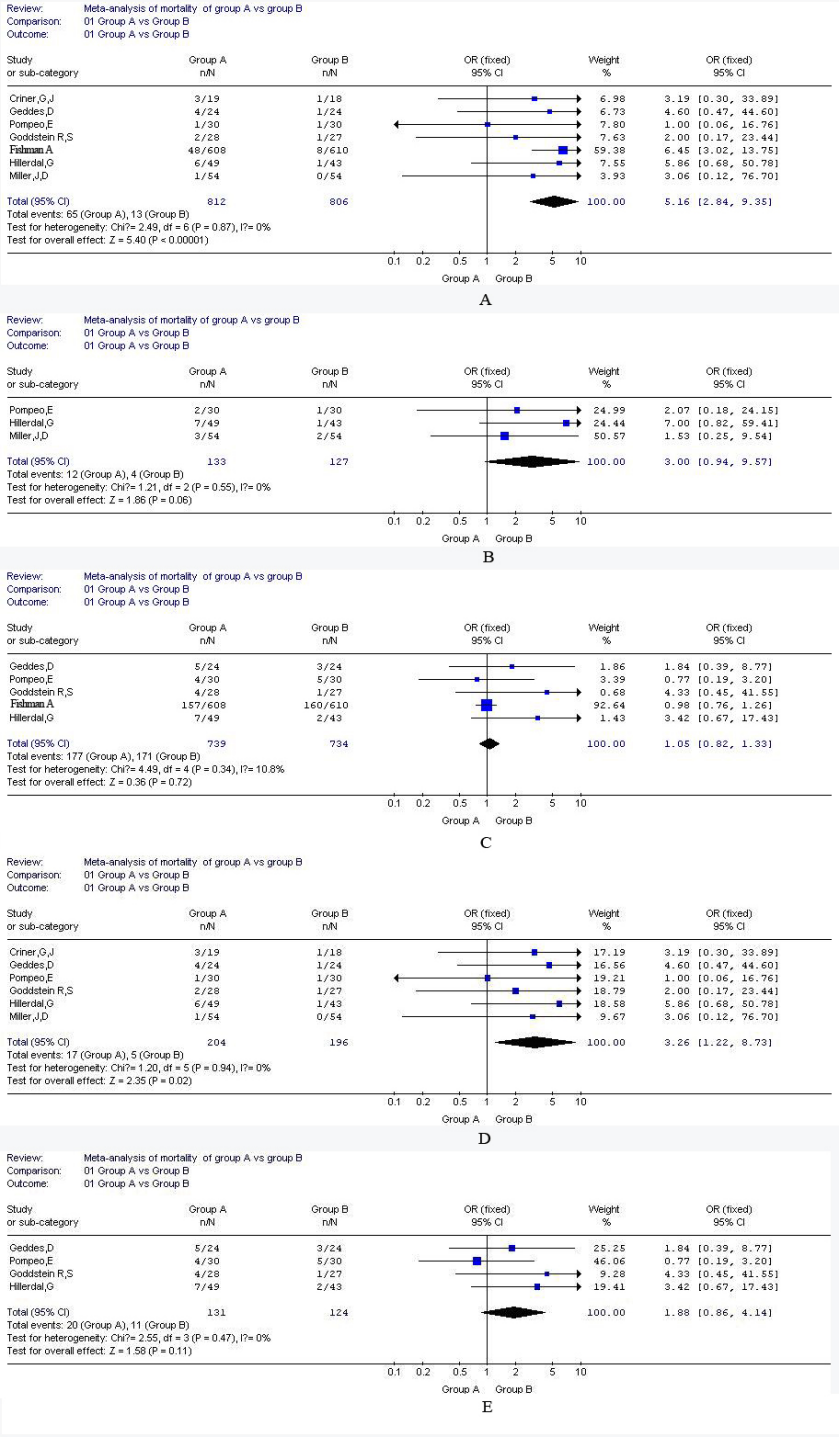
**Meta-analysis of mortality of group A and group B in 3,6 and 12 months**. A: mortality in 3 months (including Fishman's study); B: mortality in 6 months; C: mortality in 12 months(including Fishman's study); D mortality in 3 months (excluding Fishman's study); E mortality in 12 months (excluding Fishman's study).

The two trial assessors agreed upon the identified and selected RCTs. RCTs quality scores range from 3 to 5(5-point scale). Trial assessor agreement on quality assessment was strong (100%). Odds ratio (95% CI) for mortality, weighted mean difference for lung function (FEV1,FEV1%,RV%,TLC%),gas exchange(PaO2,PaCO2), DLCO% and 6MWD were depicted in Figures 1A-E and Table 2.

**Table 2:** A summary of lung function, gas exchange, DLCO% and 6MWD of five RCTs on the 3^rd^, 6^th^, 12^th ^postoperative month

Variables	Postoperative follow-up (month)	No of RCTs	No of total patients	GroupA/B	WMD95%CI	p-value
	3	2[12,16]	80	39/41	0.23(0.08,0.37)	0.002
FEV1	6	3[2,17,19]	180	89/91	0.32(0.23,0.41)	<0.00001
	12	3[2,17,19]	169	65/64	0.28(0.20,0.36)	<0.00001
	3	2[12,16]	80	39/41	11.31(6.29,15.69)	<0.00001
FEV1%	6	3[2,17,19]	180	89/91	10.16(7.42,12.89)	<0.00001
	12	3[2,17,19]	167	85/82	7.65(4.97,10.33)	<0.00001
	3	2[12,16]	80	39/41	-54.44(-75.23, -33.85)	<0.00001
RV%	6	3[2,17,19]	170	87/83	- 54.09( - 64.66, -43.52)	<0.00001
	12	3[2,17,19]	166	86/80	- 53.42( - 63.74, -43.10)	<0.00001
TLC%	3	2[12,16]	80	39/41	- 21.70( - 30.98, -12.42)	<0.00001
	6	2[2,19]	115	59/56	- 15.73( - 22.44, -9.02)	<0.00001
	12	3[2,17,19]	120	59/61	- 16.24( - 23.07, -9.41)	<0.00001
DLCO%	6	2[17,19]	105	52/53	0.01(-0.25,0.27)	0.95
	12	2[17,19]	96	51/45	0.01(-0.25,0.27)	0.25
Pa02	6	2[17,19]	136	67/6	9.98(9.65,10.13)	<0.00001
	12	2[2,17]	114	60/54	6.37(6.10,6.64)	<0.00001
PaC02	6	4[2,11,17,21]	229	108/121	- 1.54(- 1.72, - 1.36)	<0.00001
	12	2[2,17]	114	60/54	-2.00(-2.23, -1.77)	<0.00001
	3	2[12,16]	80	39/41	10.5(-16.30,37.38)	0.44
6MWD	6	5[2,11,17,19 ,21]	274	129/145	68.34(36.58,100.09)	<0.00001
	12	3[2,17,19]	168	85/83	76.92(60.87,92.98)	<0.00001

### Meta-analysis of mortality of the group underwent LVRS (group A, similar thereinafter) and the group received conservative medical therapies (group B, similar thereinafter) in 3,6 and 12 months

Odds ratio (95%CI), expressed as the mortality of group A versus group B, was 5.16(2.84, 9.35) in 3 months, 3(0.94, 9.57) in 6 months, 1.05(0.82, 1.33) in 12 months, respectively.

Figure 1 A demonstrated that mortality in 3 months was significantly lower in group B than in group A (test for overall effect, Z = 5.4, *P *< 0.0001), as well as the same trend on mortality in 6 months despite no statistical significance (Z = 1.86, *P *= 0.06) (Figure 1B). However, there was no significant difference of mortality between the two groups in 12 months (Figure 1C, test for overall effect, Z = 0.36, *P *= 0.72). Additionally, we got the same trend regarding the mortality in either 3 or 12 months (Figure 1D and 1E, Z = 2.35, *P *= 0.02; Z = 1.58, *P *= 0.11, respectively) after excluded the results from Fishman's study which was an extremely large sample trial and might result in the bias.

### Meta-analysis of lung function, gas exchange and DLCO% a nd 6MWD on the 3^rd^, 6^th ^and 12^th ^month

We conducted meta-analysis from six RCTs regarding lung function, gas exchange and DLCO% and 6MWD on the 3^rd^, 6^th^, 12^th ^month (Table 2). Two RCTs (Fishman A et al. [18] and Geddes et al [12]) were not included without expression of "mean ± SD" of aforesaid indices.

On the 3^rd^, 6^th ^and 12^th ^month, all lung function indices of group A were improved more significantly compared with group B (Table 2). PaO2 and PaCO2 on the 6^th ^and 12^th ^month were available in the RCTs, and either showed the same trend (Table 2). 6MWD of group A on the 6^th ^month and 12^th ^month were improved significantly than of group B (Table 2), although there was no difference on the 3^rd ^month.

### Systematic review of QOL

The RCTs strongly suggested that QOL of group A was better than of group B in the 6 and 12 months [2,12,16-21] (Geddes's study [12] indicated no difference between the groups in 3 months). Besides, long-term follow up also supported the aforementioned conclusion [18,20] (Mineo et al. [20]: 48 months and Fishman A et al. [18]: 24 months).

### Comparison of two surgical approaches (MS vs VATS)

Only one RCT [13] regarding comparison of MS and VATS indicated (1) there was no difference of 90-day or overall mortality (*P *= 0.67 and 0.42, respectively), (2) mean intra-operative blood loss or transfusion needs were similar (*P *= 0.99), (3) mean operation time of MS was shorter 21.7 minutes in comparison with VATS (*P *< 0.001), (4) intra-operative complications and hypoxemia of MS was less in comparison with VATS (*P *= 0.02, *P *= 0.004), (5) hospital stay of post LVRS was longer for MS than VATS (*P *= 0.01), (6) at postoperative 30 days, independently living patients were less for MS than VATS (*P *= 0.02), (7) there was no appreciable difference in lung function between the two approaches after follow-up 12 and 24 months, (8) costs for either operation or the associated hospital stay were less for VATS than for MS (*P *< 0.01).

### Clinical efficacy of using bovine pericardium for buttressing, autologous fibrin sealant and BioGlue during LVRS

In 2000, a RCT [23] was conducted in 65 patients underwent bilateral lung volume reduction surgery by VATS, either without (control group) or with bovine pericardium for buttressing. The RCT demonstrated using bovine pericardium significantly decrease the median air leak time compared with control group (0.0 day [range, 0 to 28 days versus 4 days [range, 0 to 27 days); p < 0.001), as well as median drainage time (5 days [range, 1 to 35 days] versus 7.5 days [range, 2 to 29 days); *P = *0.045).

In 2008, a RCT [15] was conducted in 25 patients undergoing bilateral LVRS by VATS. The result indicated that mean value of the total severity scores of air leak for the first 48 hours postoperative was significant lower in the treated side (with using autologous fibrin sealant) than in the control side (without using autologous fibrin sealant) (*P *< 0.01), independently of the length of the resection. Prolonged air leak and mean duration of drainage were also significantly reduced in the treated group compared with the control group (4.5% and 2.8 ± 1.9 days versus 31.8% and 5.9 ± 2.9 days, respectively)(*P *= 0.03, *P *< 0.01).

In 2009, a pilot RCT [14] was conducted in ten patients undergoing LVRS via MS approach. Each case was treated with BioGlue on one side randomly and pericardial buttress on the other side as an adjunct to the staple line. The result suggested that BioGlue treated side had the shorter mean duration of air-leak (3.0 ± 4.6 vs 6.5 ± 6.9 days), lesser chest drainage volume(733 ± 404 vs 1001 ± 861 ml) and shorter time to chest drain removal (9.7 ± 10.6 vs 11.5 ± 11.1 days) compared with pericardial buttress side.

## Discussion

World Health Organization suggested that emphysema will probably become the third cause of death with cigarette smoking [24]. More and more studies are focusing on the treatment of emphysema which is still untoward. Conservative medical therapies can not provide satisfactory long term therapeutic efficacy [5]. With regard to LVRS, the mortality and efficacy is still controversial. Therefore, we deem it essential to synthesize the published RCTs, evaluate safety, assess short-term efficacy and long-term sequel of LVRS by systemic review and meta-analysis.

Meta-analysis suggested that postoperative mortality of LVRS group gradually decreased from 3 months to 12 months. In the initial three months, the mortality of LVRS group was significantly higher than conservative treatment group due to respiratory failure and pulmonary infection [2,12,16-21]. However, there was no significant difference on mortality between the two aforesaid groups until the 12 months. NETT [25] suggested that the high risk factors of LVRS, including FEV1 <20% of predicted value with either homogeneous emphysema or DLCO% <20%, lead to higher thirty-day and overall mortality. In addition, Fishman et al. [18] suggested mortality in three months following LVRS was lower in the patients suffering from upper-lobe emphysema combining low exercerise capacity even compared with conservative treatment group, probably due to clearer target areas or more accessible areas for surgical excision. Intriguingly, Fishman et al. [18] demonstrated that overvall mortality of LVRS group (except high risk patients) was statistically lower than of conservative treatment group after 5 years follow up.

Meta-analysis also suggested lung function indices of LVRS group were appreciably improved on the 3^th^, 6^th ^and 12^th ^postoperative month, compared with conservative treatment group, in accordance to results of Fishman's study [18]. With regard to DLCO %, there was not appreciablly difference between two groups (*P *> 0.05), consisting with outcomes of Geddes's study [12]. However, only two RCTs mentioned DLCO %, and more data should be accumulated.

The effect of LVRS on PaO2 and PaCO2 was still controversial. For instance, Geddes's study [12] demonstrated insignificant improvment of PaO2 and PaCO2 following LVRS. Snyder's study [26] manifested that LVRS can increase PaO2 efficaciously (*P *> 0.001)(PaCO2 not shown). In addtion, Cremona et al. [27]suggested that LVRS improved PaO2, without significant effect on PCO2. However, our meta-analysis demonstrated that LVRS can increase PaO2 and decrease PaCO2 appreciably.

With respect to 6MWD, we concluded that LVRS can obviously increase exercise capacity on the 6^th ^and 12^th ^postoperative month, in accordance to outcomes of Fishman's [18] and Geddes's study [12]. Naunheim et al. [28] reported that improvement of post-operative exercise capacity can be maintained up to 3 years, especially in upper-lobe emphysema combining low exercise capacity.

Despite the different assessment criterion of the seven included RCTs, all the studies concluded that LVRS can improve QOL efficaciously. Krachman et al. [29] found that in patients with severe emphysema, LVRS, but not continued optimal medical therapy, results in improved sleep quality and nocturnal oxygenation. Kozora et al. [30] found that LVRS group demonstrated improvement in specific neuropsychological functions, depression, anxiety compared with conservative treatment group due to the unclear mechanisms. Additionally, compared with medical therapy, LVRS reduces the frequency of COPD exacerbations, increases the time to first exacerbation, and has a significant effect on the composite QOL survival endpoint tested [31,32].

The published RCT [13] concluded that morbidity and mortality were similar following either MS or VATS, as well as pulmonary function indices. Additionally, another study [33] suggested that two techniques offered similar outcomes regarding postoperative pain and complications, but VATS allowed earlier recovery at a lower cost than MS.

A variety of biologic and synthetic materials have been utilized to prevent and minimize air leak, including buttressing materials for instance bovine pericardium, poly-tetrafluoroethylene(PTFE), Teflon, polyglycollic acid and gel foam [14,15,23]. In patients suffering from severe emphysema and undergoing LVRS, buttressing of staple lines with bovine pericardial strips are traditionally and routinely used to reduce air leaks from rarefied emphysematous lung tissue. However, its efficacy remains inconclusive. Hazelrigg's study [34] suggested that using buttressing reduced 2 to 3 days of hospital stay. And Stammberger's RCT [23] indicated a statistically significant reduction in air leak duration after bilateral, buttressed, thoracoscopic LVRS, in spite of no reduction in length of stay. However, Moser et al. [15] pointed that buttressing adjuncts might result in the extensive inflammatory reaction with formation of dense adhesions, which enhances difficulty of lung transplantation after LVRS. And Moser et al. [15] suggested that autologous fibrin sealant overcomes the potential infective and antigenic risk in comparison to using pooled human fibrinogen or exogenous thrombin, especially of bovine origin. Besides, the results of Rathinam's study [14] also prefer BioGlue to conventional buttresses in terms of reduction in air-leak, chest drainage volumes, duration of chest drainage and significant absence of complications. Recently, a new rigid applicator tip (CryoLife Inc, U.S.A), 34 cm in length, is developed and enables precise delivery in VATS. We deem BioGlue and autologous fibrin sealant the efficacious methods to reduce air leak following LVRS.

Currently, a variety of novel, less invasive bronchoscopic lung volume reductions (BLVR) has proceeded to human trials including one-way occlusive valves, sclerosant biogels, and endobronchial bypass tract formation[10,35]. However, current data available in the small, nonrandomized studies only showed short-term improvement of dyspnoea and quality of life post BLRV [10]. Thus, further RCTs are required regarding the comparison of BVRL and LVRS [36].

Collectively, LVRS offers the more benefits regarding survival, lung function, gas exchange, exercise capacity and QOL, despite high postoperative mortality in initial three months. As a result, LVRS should be duly recommended to patients with severe emphysema and without high risk factors. With regard to LVRS approaches, we prefer VATS to MS, due to the earlier recovery and lower cost. And we deem autologous fibrin sealant and BioGlue an efficacious method to reduce air leak following LVRS.

## Abbreviation

LVRS: (Lung volume reduction surgery); RCTs: (randomized controlled trials); VATS: (video-assisted thoracoscopic surgery); MS: (median sternotomy); FEV1: (forced expiratory volume in one second); RV: (residual volume); TLC: (total lung capacity); PaO2: (pressure of arterial partial oxygen); PaCO2: (pressure of arterial CO2); DLCO: (Diffusion capacity of the lung for carbon monoxide); 6MWD: (six-minute walk distance); QOL: (Quality of life); FEV1: (forced expiratory volume in one second); FEV1%: (percentage of predicted values for FEV1); RV%: (percentage of predicted values for residual volume); TLC%: (percentage of predicted values for total lung capacity);

## Competing interests

The authors declare that they have no competing interests.

## Authors' contributions

BD and WH carried out to study design and RCTs assessment. WH, RWW and BD carried out data analysis and manuscript writing. QYT, YGJ, YGL, JHZ and YH participated in writing the manuscript. All authors read and approved the final manuscript.
